# Transmembrane voltage regulates binding of annexin V and lactadherin to cells with exposed phosphatidylserine

**DOI:** 10.1186/1471-2091-10-5

**Published:** 2009-02-17

**Authors:** Christina Smith, Donald F Gibson, Jonathan F Tait

**Affiliations:** 1 Department of Laboratory Medicine, University of Washington, Seattle, WA 98195-7110, USA

## Abstract

**Background:**

Cells expose phosphatidylserine during apoptosis. The voltage across the plasma membrane also decreases or disappears during apoptosis, but the physiological significance of this is unknown.

**Results:**

Here we show that transmembrane potential regulates membrane binding of two unrelated proteins that recognize exposed phosphatidylserine on apoptotic cells. In Jurkat T leukemia cells and K562 promyelocytic leukemia cells undergoing apoptosis, extracellular binding of annexin V was increased by decreasing membrane potential in a dose-dependent manner. Studies with phospholipid vesicles showed that the effect was mediated via an increase in binding affinity. The effect was independent of the apoptotic stimulus. The same phenomenon occurred with lactadherin, a structurally unrelated protein that also binds to apoptotic cells via phosphatidylserine and is essential for in vivo clearance of dying cells.

**Conclusion:**

Alterations in membrane potential regulate the binding of annexin V and lactadherin to cell membranes, and may also influence the membrane binding of other classes of phosphatidylserine-binding proteins.

## Background

During apoptosis, many biochemical changes occur to prepare the cell for death and removal [1]. One well-known change is the exposure of phosphatidylserine (PS) early in apoptosis, which is one signal that triggers phagocytosis of cells and cell fragments [2]. Exposed PS is recognized both by soluble proteins such as annexins [3,4] and lactadherin [5], and by membrane receptors on phagocytic cells [2,6]. This implies a complex regulatory system to mark certain cells for phagocytosis while sparing others. Annexin V is also widely used as an experimental tool to detect PS exposure both in vitro and in vivo [7-9]. Thus, factors regulating the interaction of PS-binding proteins with cells are likely to be important in both the biological effector functions of these proteins, and their use as imaging and targeting agents in experimental and diagnostic studies.

Cells also become depolarized during apoptosis. There is evidence that plasma membrane potential, in addition to mitochondrial membrane potential, is decreased early in apoptosis [10,11]. Although this phenomenon is well documented, its physiologic significance is unclear [12]. One possible role would be to regulate the binding of annexins and other PS-binding proteins to apoptotic cells. In 1997 Hoffmann *et al*. showed that the binding of annexin V to artificial phospholipid bilayers could be modulated by transmembrane potential [13]. However, this work involved application of large voltages (up to 200 mV) in an artificial system, so it is unclear whether this phenomenon would also occur in the much more complex milieu of natural cell membranes with their more modest transmembrane potentials (typically 70 mV or less). Our goal was to see if this occurred in more physiologic systems. We find that in Jurkat T leukemia and K562 promyelocytic leukemia cells undergoing apoptosis, extracellular binding of annexin V increases as membrane potential decreases. Unexpectedly, the same effect was observed with a structurally unrelated PS-binding protein, lactadherin. This indicates that membrane potential may regulate the cellular binding of PS-binding proteins in general.

## Methods

### Proteins and cell lines

Cell lines were from the American Type Culture Collection: Jurkat T leukemia clone E6-1 (ATCC TIB-152) and chronic myelogenous leukemia line K562 (ATCC TIB-1520). Recombinant annexin V-117, annexin V-128 and annexin V-137 [14] were labeled on their single N-terminal cysteine residues with IAF or AlexaFluor680 C_2 _maleimide (Invitrogen) [15]. Lactadherin (mouse) from R&D Systems was labeled with FITC as described [16].

### Treatments to induce apoptosis and alter membrane potential

Flasks of cells were given fresh media, exposed to 302-nm UV light for 7.5 min at room temperature, and then put back in the incubator for 3.5 h. Apoptosis was also induced with cycloheximide (100 μM), staurosporine (2.5 μM) or actinomycin D (10 μM) as described for Jurkat cells [7]. After treatment, cells were collected by centrifugation and suspended in assay buffer. Most assays were performed in "A buffer": 10 mM HEPES-Na pH 7.4, 130 mM NaCl, 4 mM KCl, 0.9 mM MgCl_2_, 0.8 mM NaH_2_P0_4_, 5 mM glucose, 1 mg/ml BSA, and unless noted, 1.25 mM CaCl_2_. Membrane potential was altered with a high-potassium "B buffer", with the same composition as A buffer except for 4 mM NaCl and 130 mM KCl. The potassium ionophore valinomycin and the monovalent cation ionophore gramicidin were used at 1 μM final concentration to alter membrane potential.

### Flow cytometric measurements of protein binding and membrane potential

Flow cytometry assays were set up with 2.5 to 3.0 × 10^6 ^cells/ml in A or B buffer with 30 nM AlexaFluor680-annexin V-117 and 65 nM of the anionic potentiometric probe, DiBAC_4_(3) (Molecular Probes, B438). In some experiments, DiBAC_4_(3) was omitted and assays were performed with either 30 nM IAF-annexin V-117 or 20 nM FITC-lactadherin. After incubation in the dark at 25°C for 6 min, cells were analyzed on a flow cytometer with a 488-nm laser. The cells were delineated with forward and side-scatter gating; DiBAC_4_(3) was read in channel FL1 and AlexaFluor680-annexin V-117 was read in channel FL3. FITC and IAF were read in channel FL1. Control experiments showed that the same results were obtained for cells incubated at 37°C during the annexin V binding step.

### Calcium titrations of phospholipid vesicles to determine binding affinity

We used a fluorescence assay to measure the binding of IAF-annexin V-128 to phospholipid vesicles labeled with rhodamine; as the fluorescent protein binds, the fluorescein fluorescence is progressively quenched by resonance energy transfer, allowing the fraction of bound protein to be calculated from the observed quenching divided by the maximum quenching at saturation [15]. Unilamellar phospholipid vesicles were prepared [16] with 25% PS (1-palmitoyl,2-oleoyl), 2% rhodamine-labeled phosphatidylethanolamine, 20% 1,2-diheptanoyl-phosphatidylcholine and 53% 1-palmitoyl,2-oleoyl phosphatidylcholine (all from Avanti Polar Lipids, Alabaster, AL). Vesicles were prepared in a buffer containing 50 mM HEPES-sodium, pH 7.4, 3 mM NaN_3_, and either 1) 100 mM NaCl; or 2) 99 mM NaCl plus 1 mM KCl; or 3) 100 mM KCl. Assays were performed with vesicles diluted into one of these three buffers to give various combinations of potassium gradients between the inside and the outside of the vesicle. Reactions contained 1 nM IAF-annexin V-128, 10 μM phospholipid vesicles, either 0 or 1 μM valinomycin, and various concentrations of calcium chloride. After a 10-min incubation at 25°C, fluorescence intensity was measured and fluorescence quenching calculated relative to the fluorescence intensity seen in the absence of calcium.

Equilibrium binding affinities are reported as the value of pK_d _(the negative logarithm of the dissociation constant), which was obtained from the following model [14,15,17,18]:

(1) *n *Ca + Protein + Membrane ↔ Protein*Ca_n_*Membrane

(2) K_d _= [Ca]^n ^[Membrane] [Protein]/[Protein*Ca_n_*Membrane]

(3) pK_d _= -log K_d _= -(n log EC_50 _+ log [Membrane])

The EC_50 _(midpoint of titration curve) and n (slope of titration curve) values were determined by non-linear least-squares fitting of the calcium titration curves to the following function:

(4) Q/Q_max _= [Ca]^n^/([Ca]^n ^+ EC_50 _^n^)

where Q is the observed fluorescence quenching at a given calcium concentration, and Q_max _is the maximum quenching observed when all fluorescent protein is bound to the vesicles. The apparent dissociation constant of protein for membrane at a constant calcium concentration can be estimated from [15]:

(5) K_d, app _= K_d_/[Ca]^n^

Under the assay conditions used here, the pK_d _for binding to vesicles with 25% PS is about 39 in the absence of a transmembrane voltage gradient [18]. Due to the negative cooperativity of binding [17], all these quantitative analyses are only applicable at low membrane occupancy, typically below 10% of the level obtained when the membrane is fully saturated with protein. Under the assay conditions used in this study, the phospholipid vesicles are only 1% saturated when all the added annexin V is bound.

## Results

### Transmembrane voltage regulates annexin V binding affinity for phospholipid vesicles

We first sought to confirm the findings of Hofmann *et al. *[13] in a phospholipid vesicle system that is closer to physiological conditions. We used the potassium-selective ionophore valinomycin to create positive or negative transmembrane diffusion potentials in the presence of KCl concentration gradients between the outside and the inside of vesicles containing 25% PS (Table 1). When valinomycin is added, the binding affinity (pK_d_) of annexin V for the external face of the membrane increases when the inside of the vesicle is made more positive (inward gradient of K^+^). Likewise, the binding affinity decreases by about the same amount when the inside of the vesicle is made more negative (outward gradient of K^+^), confirming the expected symmetry of the effect.

**Table 1:** Transmembrane voltage regulates annexin V binding to phospholipid vesicles.

[K^+^] outside (mM)	[K^+^] inside (mM)	Valino-mycin	Number of assays	EC_50 _(mM)	Slope	pK_d _(experimental)	pK_d, app _at 1.25 mM Ca^2+ ^(theoretical)
100	0	-	10	0.113 ± 0.004	8.7 ± 0.2	41.5 ± 1.0	16.2
100	0	+	11	0.116 ± 0.005	10.1 ± 0.4	46.6 ± 1.5	17.2
						
					Difference:	+5.1*	+1.0
1	100	-	4	0.135 ± 0.003	9.2 ± 0.2	42.5 ± 0.7	15.8
1	100	+	3	0.132 ± 0.004	8.1± 0.1	38.5 ± 0.3	15.0
						
					Difference:	-4.0*	-0.8

Binding of fluorescein-annexin V-117 to phospholipid vesicles containing 25% PS was measured by calcium titration at low membrane occupancy [15] and binding parameters EC_50_, slope, and pK_d _(log of binding affinity) were determined as described under Methods. A positive change in pK_d _indicates higher-affinity binding. Assays were performed with the indicated concentrations of KCl outside and inside the vesicles; a transmembrane diffusion potential was created by the addition of 1 μM valinomycin, a potassium-selective ionophore. Uncertainties on experimental parameters are given as SEM; asterisks indicate differences that are statistically significant (*p *< 0.02) by two-tailed *t *test. The theoretical apparent pK_d _at 1.25 mM calcium chloride was calculated from Equation 5.

The pK_d _values determined in Table 1 represent the free energy change that occurs in going from completely calcium-free protein and phospholipid to the final bound complex of protein-calcium-phospholipid (Equation 1 in Methods). Under physiologic conditions, some calcium is already bound to both protein and phospholipid before the protein-membrane binding event occurs, and therefore the incremental free energy change for the protein-binding step will be less than for the entire reaction scheme given in Equation 1. One can estimate an apparent dissociation constant for the protein-binding step at a constant calcium concentration from Equation 5 [15]. To estimate how large the effect of transmembrane potential might be under physiologic conditions, we estimated a K_d, app _that would pertain to protein-membrane binding interactions occurring at a constant free calcium concentration of 1.25 mM. The theoretical calculation indicates that the apparent K_d _will increase by about a factor of 10 (ΔpK_d _of 1.0) (see rightmost column in Table 1). How much this will alter annexin V binding depends in turn on the apparent affinity for annexin V, and the concentration of annexin V used in the assay. The affinity of annexin V for cells is much less than for the phospholipid vesicles with 25% PS used in Table 1, with pK_d _values around 30 [15] and apparent K_d _values reported in the range from about 5 to 30 nM [19-21].

**Figure 1:**
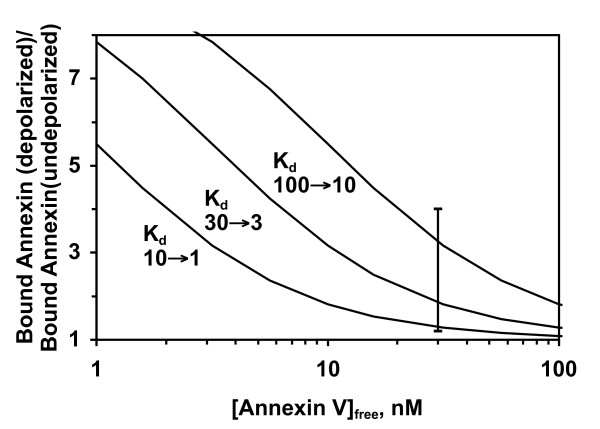
**Theoretical calculation of how increased binding affinity due to depolarization could increase annexin V binding under different conditions of affinity and ligand concentration**. Five theoretical binding isotherms were calculated for K_d _values of 1, 3, 10, 30, and 100 nM from the Langmuir isotherm (bound fraction = [annexin V]/([annexin V] + K_d_)). These curves were then used to calculate the effect of a ten-fold increase in binding affinity (ΔpK_d _of +1) on the relative amount of annexin V bound to a cell for three different situations: K_d _changes from 10 nM to 1 nM (left curve); 30 to 3 nM (middle curve); 100 to 10 nM (right curve). The K_d _range from 1 to 100 nM was chosen for illustration because experimental apparent K_d _values for annexin V binding to cells are in this general range [19-21]. The *vertical bar *indicates the experimentally observed range of depolarized/undepolarized binding ratios under the various experimental conditions used in this study (see subsequent figures).

Figure 1 shows a family of theoretical curves for the situation where the K_d, app _becomes tenfold tighter as a result of changes in the membrane potential. When the concentration of annexin V is far below the apparent K_d_, the effect of membrane potential can be very large (as much as a ten-fold increase in binding), but as the concentration of annexin V increases above the apparent K_d_, the relative increase in binding becomes smaller and smaller. Although this theoretical analysis does not take account of all the complexities of binding to natural cell membranes, it does provide a general guide to the magnitude of the effect that might be observed with living cells under physiologic conditions.

### Depolarization increases binding of annexin V to cells undergoing apoptosis

**Figure 2:**
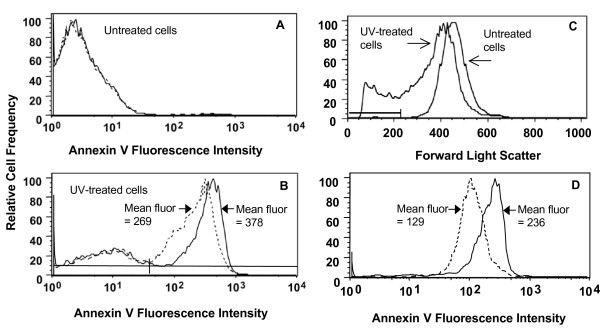
**Depolarization increases binding of annexin V to cells with exposed PS**. Jurkat cells were left untreated (Panel A) or were exposed to UV light to induce apoptosis (Panel B). After 3.5 h, cells were assayed by flow cytometry with fluorescein-annexin V-117. Most of the UV-treated cells have entered the apoptotic pathway and have exposed PS on the extracellular face of the plasma membrane, as indicated by the development of a sizable population of annexin-positive cells. Depolarization with high-potassium Buffer B (solid lines) increases the mean fluorescence intensity of annexin-positive cells about 41% compared to results obtained in low-potassium Buffer A (dashed lines). However, depolarization does not alter the mean fluorescence of the annexin-negative population in either untreated or UV-treated cells. The horizontal bars in the lower part of Panel B indicate the gates used to define the annexin-negative and annexin-positive cell populations to allow calculation of mean fluorescence intensities for these cell populations. Panel C: Forward-scatter histograms for untreated cells or UV-treated cells. The horizontal line indicates the gate used to define the shrunken cells that are observed only in apoptosis. Panel D: Annexin V histograms for the shrunken apoptotic cells assayed in low-potassium A buffer (dashed lines) or high-potassium B buffer (solid lines). Depolarization increases annexin V binding to this population by about 83%.

We next tested whether living cells would show altered annexin V binding as a function of changes in membrane potential. Jurkat T leukemia cells provide a good model system, as they normally have a significant resting membrane potential of about -60 mV (i.e., outside positive and inside negative) [11] and also expose PS early in apoptosis [7]. When Jurkat cells are treated with UV light, they start to undergo apoptosis, as shown by the development of a population of annexin-positive cells (Figure 2, Panel B versus Panel A). Depolarization with high-potassium buffer (Panel B, solid line versus dotted line) increases the average annexin V binding of the annexin-positive population, but does not alter the percentage of annexin-negative cells in either untreated or UV-treated cells. These results are as predicted from the vesicle binding studies above, i.e. making transmembrane potential less negative would increase the binding of annexin V to the external face of the plasma membrane. The magnitude of the change in binding is also reasonably consistent with the theoretical predictions of Figure 1: at an annexin V concentration of 30 nM and a starting K_d, app _of 10 nM, Figure 1 predicts an increase in binding of about 1.3-fold, consistent with the observed value of about 1.4.

Cells shrink as they progress through apoptosis, and flow cytometry can detect this change via a decrease in forward-scatter light intensity (Figure 2C). The subpopulation of apoptotic cells with the lowest average size showed the greatest relative increase in annexin binding with depolarization (Figure 2D): the mean fluorescence intensity increased by 83% for this subpopulation, versus 41% for the entire annexin-positive population in Panel B.

**Figure 3:**
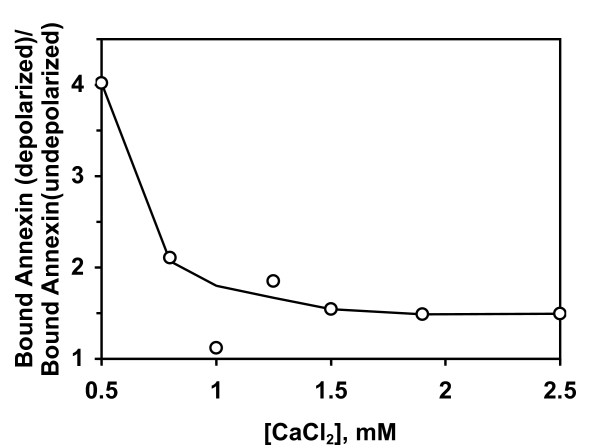
**Depolarization causes larger relative increases in annexin V binding at lower calcium concentrations**. Jurkat cells were treated with UV light, then assayed by flow cytometry 3.5 h later. Cells were assayed with Alexa-680-annexin V-117 in either Buffer A or Buffer B with the indicated concentration of calcium chloride. The subpopulation of small apoptotic cells was selected with a forward-scatter gate as shown in Figure 2C, and the mean annexin V fluorescence of this population was calculated. The graph shows the ratio of mean annexin V fluorescence in depolarizing buffer to mean annexin V fluorescence in non-depolarizing buffer at each calcium concentration.

Figure 1 also predicts that assays performed under conditions that lower the average affinity of binding should show a relatively larger effect of depolarization on the binding of annexin V. To test this, we performed assays at different calcium concentrations, which will vary the K_d, app _(Equation 5). As shown in Figure 3, the relative increase in annexin V binding due to depolarization becomes greater as binding affinity decreases at lower calcium concentrations, consistent with the predictions from Figure 1.

**Figure 4:**
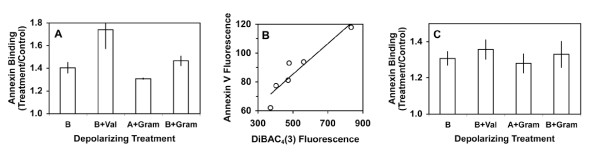
**Effect of depolarizing agents on annexin V binding to apoptotic Jurkat and K562 cells**. Panel A: Jurkat cells were treated with UV light; 3.5 h later, cells were assayed in one of the following buffers: buffer A; buffer B; buffer A + 1 μM gramicidin; buffer B + 1 μM gramicidin; buffer B + 1 μM valinomycin. Results are expressed as the mean fluorescence of the annexin-positive cells for each treatment relative to the mean fluorescence of cells assayed in non-depolarizing A buffer. Results are mean ± SEM for two to nine independent experiments for each treatment; all treatment/control ratios are significantly different from 1.0 by two-tailed *t *test (*p *< 0.02). Panel B: Correlation between increased annexin V binding and degree of depolarization as measured with the membrane-potential-sensitive dye DiBAC_4_(3). Jurkat cells treated as described in Panel A were assayed by two-color flow cytometry with DiBAC_4_(3) and AlexaFluor680-annexin V-117. DiBAC_4_(3) uptake increases as membrane potential becomes less negative. Panel C: K562 cells were treated with UV light and then assayed as described in Panel A. Results are mean ± SEM of two to four independent experiments; all treatment/control ratios are significantly different from 1.0 by two-tailed *t *test (*p *< 0.02).

To further characterize the effect of depolarization, we tested several different depolarizing treatments (Figure 4). Treatment of apoptotic Jurkat cells with various combinations of high-potassium buffer, valinomycin (a potassium-selective ionophore) or gramicidin (a non-specific ionophore for both sodium and potassium) all caused increased annexin V binding (Figure 4, Panel A). The relative increase in annexin V binding correlated with the degree of membrane depolarization as measured by the membrane-potential-sensitive dye DiBAC_4_(3) (Panel B). We also tested a second cell line: the promyelocytic leukemia cell line K562 showed a similar increase in annexin V binding in response to the same treatments (Panel C). We also verified that the observed effect was not limited to UV-treated Jurkat cells. We observed the same pattern of increased annexin V binding in response to depolarization with high-potassium buffer for Jurkat cells treated with cycloheximide (annexin binding ratio of 1.20 ± 0.02, mean ± SEM, n = 4 experiments), staurosporine (1.17, single experiment) and actinomycin D (1.24, single experiment).

Several controls were done to rule out potential alternative explanations for the observed effect. To verify that treatment with high-potassium buffer was not by itself increasing PS exposure, we showed that transient preincubation in high-potassium B buffer did not increase annexin V binding of cells subsequently assayed in low-potassium A buffer. Decreased membrane potential increased the binding of annexin V labeled with different fluorophores (fluorescein and AlexaFluor680) attached to either amino groups or the N-terminal cysteine, indicating that the change in fluorescence signal was not due to effects on the fluorophore *per se*. Experiments with a mutant form of annexin V (annexin V-137 [14]) that lacks PS binding activity showed no binding to normal or apoptotic cells in either A or B buffer, ruling out a process of non-specific uptake in dead or dying cells. The effect was observed when the assay was performed in the presence of 10% fetal calf serum, indicating that proteins typically present in the extracellular milieu would not mask PS binding sites in vivo. The same effect was seen when cells were labeled with annexin V at either 22° or 37°, and over a range of annexin V and calcium concentrations.

### Depolarization also increases the binding of lactadherin, another PS binding protein structurally unrelated to annexin V

**Figure 5:**
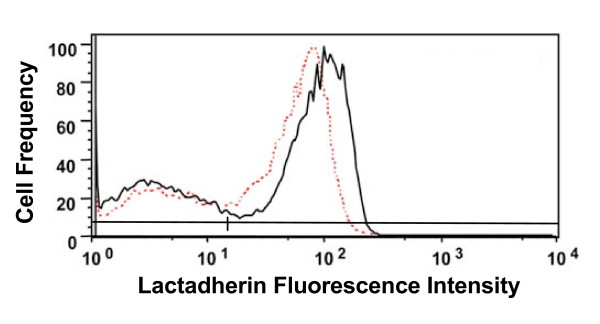
**Depolarization increases the binding of lactadherin to apoptotic cells**. Jurkat cells were prepared as described in the legend to Figure 2, and were then assayed with fluorescein-lactadherin in low-potassium A buffer (dashed lines) or depolarizing high-potassium B buffer (solid line). Assay in B buffer increased the mean fluorescence of the lactadherin-positive cell population by an average of 33 ± 2% compared to cells assayed in A buffer (mean ± SEM of three separate experiments). Horizontal lines indicate the gates used to define lactadherin-negative and lactadherin-positive cells.

To evaluate the generality of this phenomenon, we tested lactadherin, a PS-binding protein that is structurally unrelated to annexins and is known to bind to apoptotic cells [5,22,23] and promote their phagocytic clearance [5,24]. Lactadherin binds to apoptotic cells in the same manner as annexin V (Figure 5, dashed line). Depolarization of cells with high-potassium buffer (Figure 5, solid line) increased binding of lactadherin to PS-positive cells to about the same degree as for annexin V.

## Discussion

This study shows that transmembrane potential regulates the binding of PS-binding proteins to apoptotic cells. This phenomenon appears to be general, since it was observed with two structurally unrelated PS binding proteins (annexin V and lactadherin), on two different cell lines, in response to multiple apoptotic stimuli and multiple means of depolarizing cells. The effect is seen under physiologic conditions of pH, ionized calcium, and other extracellular ions. Theoretical analysis (Figure 1 and references [14,15,17,18]) predicts that the magnitude of the effect can vary greatly depending on many factors: the magnitude and sign of the membrane potential; the concentrations of calcium, annexin V, and cells or phospholipids in the assay; the percentage of PS in the membrane; and the fractional occupancy of membrane binding sites. This predicted variability is borne out in practice, as we have seen relative increases in annexin V binding anywhere from 20% to 400% in this study depending on assay conditions.

For experimental convenience, most of our experiments were performed at a relatively high concentration of annexin V (30 nM), which will tend to decrease the magnitude of the observed effect (*cf. *Figure 1). In vivo, the extracellular concentration of annexin V is usually far lower than this (0 – 10 pM in human plasma samples [25]), which would tend to magnify the relative effect of membrane potential on the binding of annexin V. Likewise, the doses of annexin V typically given for apoptosis imaging studies in humans (0.3–0.5 mg) would result in concentrations in the extracellular fluid of < 1 nM. In addition, even more factors will come into play in vivo, such as the concentrations of potential competitor proteins, and it is therefore difficult to make quantitative predictions about the effect of membrane potential on the binding of annexin V or lactadherin to cells in vivo. Nevertheless, even relatively modest increases in cell-surface density of bound annexin V or bound lactadherin on apoptotic cells could be enough to substantially increase phagocytosis, particularly if the recognition of these proteins by their cognate receptors on neighboring phagocytic cells is nonlinear. The macrophage uptake of apoptotic Jurkat cells is nonlinear in relation to the amount of PS exposed [26], and phagocytosis of apoptotic thymocytes also shows a very steep dependence on the concentration of lactadherin added to the assay [5].

At this point, the mechanism underlying this effect is unknown. The fact that it occurs with two structurally unrelated proteins and with pure phospholipid vesicles suggests it is likely due to an effect on the phospholipid component of the cell membrane rather than on the protein *per se*. The binding affinity of annexins for membranes is very strongly influenced by the local density of PS [15,18], so the effect of membrane potential might be mediated via its effect on the mobility and/or clustering of PS. Less is known about the mechanism of lactadherin-membrane binding, but a similar process could occur as well. Since lactadherin binding is calcium-independent, this suggests that the effect of membrane potential on binding may not be mediated via calcium. It will be interesting to see if this phenomenon occurs with other families of proteins that recognize PS or other anionic phospholipids, such as C2-domain proteins like protein kinase C isozymes, and coagulation factors V and VIII. It is also unknown whether this effect will occur with proteins that bind to neutral phospholipids such as phosphatidylcholine.

Our results imply that alterations in transmembrane potential may also regulate the extracellular dynamics of annexin-membrane binding in other states besides apoptosis. Although the effects of hypoxia and ischemia on neurons are complex, one effect is substantial plasma membrane depolarization, due in part to the loss of cellular ATP required to maintain normal potassium gradients [27]. A similar phenomenon would occur in other tissues such as the heart. Thus, the observed alterations in annexin V uptake in vivo in myocardial [28], neuronal [29,30] and skeletal muscle [31] ischemia may be strongly influenced by the state of the membrane potential in addition to the level of exposed PS. This could also help explain the ready reversibility of annexin V uptake in some of these conditions [31]: restoration of normal blood and oxygen supply would allow rapid restoration of the normal transmembrane ion gradients that are required to maintain membrane potential. Reduction in annexin V binding would thus not necessarily require transmembrane transport of PS to remove exposed PS from the extracellular face of the plasma membrane.

Another intriguing possibility raised by our results is that changes in membrane potential could also regulate the *intracellular *binding of annexins. The situation at the intracellular face of the plasma membrane would be the mirror image of what is observed at the extracellular face, i.e. as a cell depolarizes and transmembrane potential becomes less negative, this would decrease binding of intracellular annexins to the intracellular face of the plasma membrane. Annexins are primarily intracellular, cytoplasmic proteins, but their attachment to subcellular membranes can vary in response to multiple stimuli [32,33]. Perhaps at least some of these effects are mediated via alterations in membrane potential.

## Conclusion

Transmembrane potential may be a regulator of membrane binding of annexins and lactadherin in both normal physiology and disease states.

## Abbreviations

Buffer A: 10 mM HEPES-Na pH 7.4, 130 mM NaCl, 4 mM KCl, 0.9 mM MgCl_2_, 0.8 mM NaH_2_P0_4_, 5 mM glucose, 1 mg/ml BSA, and 1.25 mM CaCl_2_; Buffer B: same as Buffer A except for 4 mM NaCl and 130 mM KCl; DiBAC_4_(3): bis-(1,3-dibutylbarbituric acid)trimethine oxonol; EC_50_: calcium concentration causing 50% of maximum protein-membrane binding; FITC: fluorescein 5-isothiocyanate; IAF: 5-iodoacetamidofluorescein; pK_d_: negative logarithm of equilibrium dissociation constant; PS: phosphatidylserine; SEM: standard error of the mean; UV: ultraviolet.

## Authors' contributions

CS designed and performed the experiments with cells. DG designed and performed the experiments with phospholipid vesicles. JT conceived and directed the overall study and did the theoretical analysis.
